# Exploring *Lactobacillus reuteri* DSM20016 as a biocatalyst for transformation of longer chain 1,2-diols: Limits with microcompartment

**DOI:** 10.1371/journal.pone.0185734

**Published:** 2017-09-28

**Authors:** Lu Chen, Rajni Hatti-Kaul

**Affiliations:** Division of Biotechnology, Center for Chemistry and Chemical Engineering, Lund University, Lund, Sweden; Hubei University, CHINA

## Abstract

*Lactobacillus reuteri* metabolises glycerol efficiently to form 3-hydroxypropionic acid (3-HP) and 1,3-propanediol (1,3PDO) by the same mechanism as that for 1,2-propanediol (1,2PDO) conversion to propionic acid and propanol via its propanediol utilization (pdu) pathway. Pdu enzymes are encoded by the *pdu*-operon, which also contain genes encoding the shell proteins of the microcompartment housing the metabolic pathway. In this work the selectivity and kinetics of the reactions catalysed by *L*. *reuteri* DSM20016 Pdu enzymes glycerol dehydratase (GDH), 1,3-propanediol oxidoreductase (PduQ) and coenzyme-A acylating propionaldehyde dehydrogenase (PduP), produced recombinantly, was investigated against corresponding substrates of different chain lengths. Glycerol dehydratase exhibited activity against C2-C4 polyols, with the highest activity against glycerol and 1,2-propanediol (1,2-PDO). A double mutant of the *pduC* gene of GDH (PduC-S302A/Q337A) was constructed that displayed lowered activity against glycerol and 1,2PDO but extended the substrate range upto C6-diol. The best substrate for both PduQ and PduP was 3-hydroxypropanal (3HPA), although PduP exhibited nearly 10-fold higher specific activity. The enzymes also showed some activity against C3-C10 aliphatic aldehydes, with PduP having higher relative activity. Subsequently, transformation of polyols using whole cells of *L*. *reuteri* containing the wild type- and mutated GDH, respectively, confirmed the reduced activity of the mutant against glycerol and 1,2PDO, but its activity against longer substrates was negligible. In contrast, recombinant *Escherichia coli* BL21(DE3) cells harboring the GDH variant converted diols with up to C6 carbon chain length to their respective aldehydes, suggesting that the protein shell of the microcompartment in *L*. *reuteri* posed a barrier to the passage of longer chain substrate.

## Introduction

With the expansion of global biodiesel production, the major byproduct glycerol has attracted great interest as an important platform for the biobased chemical industry [1–3]. A large number of studies have thus focused on valorisation of glycerol to produce not only the chemicals that are currently produced from fossil feedstocks but even those that are not currently available in the market, e.g. 3-hydroxypropionaldehyde (3-HPA) and 3-hydroxypropionic acid (3-HP) [4–10]. Also, utilization of different microorganisms able to metabolise glycerol to 1,3-propanediol such as *K*. *pneumoniae*, *C*. *butyricum*, and *Lactobacillus* species, or their enzymes in heterologous hosts, have shown promising results from an industrial viewpoint, due to high conversion rates and product yields [11–13].

*Lactobacillus reuteri* is a probiotic organism that possesses the metabolic pathway called the propanediol utilization (Pdu) pathway that catalyzes dehydration of glycerol to 3-HPA using glycerol dehydratase (PduCDE), and further branches to 1,3-propanediol (1,3-PDO) through 1,3-propanediol oxidoreductase (PduQ), and to 3-HP via a series of reactions catalyzed by coenzyme-A acylating propionaldehyde dehydrogenase (PduP), phosphotransacylase (PduL) and propionate kinase (PduW) [14,15]. The same pathway is utilized by *L*. *reuteri* to convert 1,2-propanediol (1,2-PDO) to propionic acid and propanol [16]. The enzymes of the pdu pathway are encoded by a *pdu* operon that also codes for the structural proteins making up the microcompartment housing the pathway [17].

Bioinformatics studies have indicated that about 17% of bacterial specicies use proteinaceous microcompartments (MCPs, typically 100–150 nm in diameter) as simple organelles to localize metabolic pathways that have toxic or volatile intermediates [18–20]. Bobik and coworkers initially showed that *Salmonella* conditionally forms Pdu-microcompartment that can sequester propionaldehyde to prevent cytotoxicity during growth in the presence of 1,2-PDO [21]. The protein shells of Pdu MCPs are thought to be composed of 9 different bacterial microcompartment domain proteins, i.e. PduA, PduB, PduB’, PduK, PduJ, PduM, PduN, PduU and PduT, for which a striking feature is that they have central pores that mediate the transport of substrates, products and enzyme cofactors between the cell cytoplasm and interior of the Pdu MCPs [22]. The current view is that most MCP domain proteins are hexagonal in shape and tile edge to edge to form extended protein sheets, which then interact with the pentameric vertex proteins to develop intricate architecture [23].

Glycerol dehydratase (GDH) is considered to play an essential role in the transformation of glycerol/1,2-PDO by producing both an electron acceptor and a metabolic intermediate. The dehydratase enzyme requires cobalamin (Vitamin B12) as a cofactor whose biosynthesis is encoded by the gene sets *cbi*, *cob* and *hem*, located close to the *pdu* operon [24]. Genetic characterization of *L*. *reuteri* show the regulatory control of the *pdu-cbi-hem-cob* cluster is based on its induction by 1,2-propanediol or glycerol [24]. The operon and its mechanism have also been reported in other lactobacilli and microorganisms including *Salmonella enterica*, *Clostridium carboxidivorans*, *Klebsiella pneumoniae* and *Enterococcus malodoratus* [12,16,21,25,26]. Basically, NADH produced during glucose metabolism is preferentially reoxidized to NAD^+^ by the reduction of 3-HPA to 1,3-PDO (when glycerol is the co-substrate) or propionaldehyde to *n*-propanol (1,2-PDO as co-substrate). An adequate supply of glycerol/1,2-PDO can thus outweigh the repression of glucose on the central metabolism. In the absence of glucose, glycerol/1,2-PDO are metabolised by *L*. *reuteri* without being used for growth, and the cofactor cycling is achieved by processing of the aldehyde intermediate via the two branches of the pdu pathway described above. This constitutes a unique naturally occurring system for co-production of an organic acid and an alcohol, as shown in case of the production of 3-HP and 1,3-PDO from glycerol [14].

**Fig 1:**
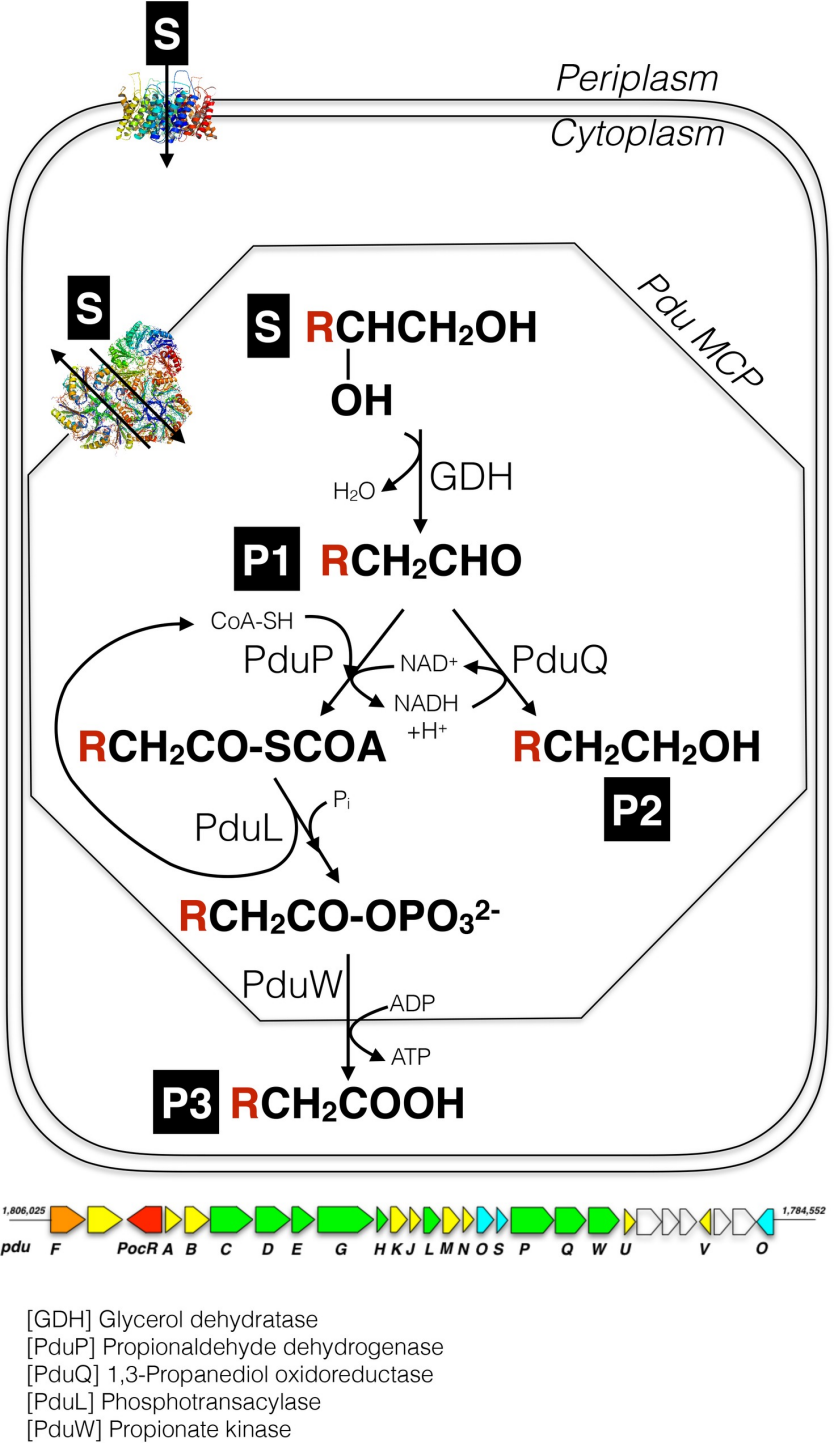
General reaction scheme for glycerol/1,2-diols conversion to corresponding alcohol and acid by lumen enzymes of *pdu*-microcompartment.

The aim of the present study was to determine how broad the scope of the substrates accepted by the key enzymes (i.e. glycerol dehydratase, PduP and PduQ) of the Pdu pathway of *L*. *reuteri* DSM20016 is, and to make use of rational engineering to increase the selectivity of glycerol dehydratase for longer chain diols, and furthermore to evaluate the combined performance of enzymes in the whole bacterial cells for transformation of the various diols (Fig 1).

## Materials and methods

### Chemicals and reagents

All restriction enzymes, IPTG and X-Gal were purchased from Fermentas. Taq DNA polymerase, and T4 DNA ligase were from New England Biolabs. BugBuster protein extraction reagent was from Novagen. MRS-medium was obtained from Beckton, Dickinson, Le Pont de Claix, France. Antibiotics, Vitamin B_12_ (CN-B_12_), coenzyme B_12_ (AdoB_12_), Coenzyme A sodium salt hydrate, NAD^+^, NADP^+^, NADH and NADPH were purchased from Sigma-Aldrich, St. Louis, Missouri, USA. All primers and kits for DNA extraction and purification were procured from ThermoFisher Scientific, while QuikChange II XL Site-Directed Mutagenesis Kit was from Agilent Technologies.

### Bacterial strains, media and growth conditions

The bacterial strains used in the study are listed in S1 Table. *L*. *reuteri* DSM20016 was initially grown in 20 mL of MRS-medium under anaerobic conditions at 37°C without shaking for 16–17 h. Cultivation was started by an inoculum (1% v/v) from a freshly grown culture grown under the same conditions. Depending on the experiment, the medium was supplemented with 20 mM glycerol (Sigma Aldrich). Cells were harvested by centrifugation at 6000 x g for 10 min and washed twice with 50 mM sodium acetate buffer, pH 5.0. Recombinant *Escherichia coli* BL21(DE3) with recombinant GDH was grown in LB medium under 37°C with shaking at 220 rpm as described below.

### Bioinformatics analysis

Genome sequence data of *Citrobacter freundii* (NZ_CP007557), *Yersinia enterocolitica* (NC_008800), *Klebsiella oxytoca* (NZ_CP011636), *K*. *pneumoniae* (NC_016845), *Salmonella enterica* (NC_003197), *Terrisporobacter glycolicus* (AUUB01.1), *Clostridium butyricum* (NZ_CP013489), *C*. *perfringens*(NC_008261), *C*. *carboxidivorans* (NZ_CP011803), *C*. *pasteurianum* (NZ_CP009268), *Lactobacillus reuteri* (NC_009513), *L*. *brevis* (NC_008497), *L*. *collinoides* (DRR017788), *Streptococcus sanguinis* (NC_009009), *Enterococcus malodoratus* (NZ_KE136481) and *Listeria monocytogenes* (NC_003210) were obtained from GenBank. An initial set of open reading frame (ORF) that probably encodes Pdu-microcompartment proteins was identified using Geneious 10.1.2 and verified against a non-redundant protein database (NCBI) using BLASTP. Multiple sequence alignment for the identification of conserved amino acids were made by ClustalW2 tools (EMBL-EBI) and visualized in Geneious 10.1.2. Homology modeling of the *L*. *reuteri* GDH was performed with the aid of Program DeepView (Swiss PDB-Viewer). Homology model quality assessment was performed by utilizing the SWISS-MODEL workspace through QMEAN4-scoring and Z-score plot. The RMS deviation value was calculated by superimposing alpha carbons of template. The tool AutoDock Vina was used as a platform for the study of dockings of GDH with glycerol and 1,2-propanediol. Figures were generated using PyMol and raytraced images were produced with POV-Ray.

### Cloning, expression and purification of wide-type/mutant glycerol dehydratase, PduP and PduQ

*L*. *reuteri* DSM 20016 was cultivated in 20 mL MRS medium under anaerobic conditions in closed vials at 37°C. The cells were harvested and genomic DNA was extracted and purified using GeneJet Genomic DNA kit (Fermentas). The gene sequences coding for GDH (PduCDE) and its reactivating factor (PduGH) were amplified by the primers listed in S1 Table. Initial denaturing at 95°C for 3 min was followed by 30 cycles of denaturing at 95°C for 30 s, annealing at 53°C for 30 s and elongation at 72°C for 1.5 min. A final elongation was carried at 72°C for 10 min. The PCR products were separated and purified by agarose gel electrophoresis and inserted directly into vector pGEM-T to construct pGEM-T-*pduCDE* and pGEM-T-*pduGH*, respectively.

Site-directed mutagenesis of GDH was performed using a QuikChange II XL Site-Directed Mutagenesis Kit. Plasmid pGEM-T-*pduCDE* was used as template. The mutagenic sense and antisense primers are listed in S1 Table. The double mutant carrying both PduC-S302A and PduC-Q337A mutations was designated pGEM-T-*pduCDE* (C-S302A/Q337A). The presence of intended mutations and the absence of unintended mutations were comfirmed by sequence analysis. The mutant plasmid pGEM-T-*pduCDE* (C-S302A/Q337A) and wild type (WT) plasmid pGEM-T-*pduCDE* were digested with the restriction enzymes *Bam*HI and *Eco*RI. The resultant 2.9-kb fragment that contains the entire *pduCDE* gene, with either double mutations or wild type, was transferred to the corresponding region of pCDFDuet to construct expression plasmids pCDFDuet-*pduCDE* (C-S302A/Q337A) and pCDFDuet-*pduCDE*, respectively. The plasmid pGEM-T-*pduGH*, containing the genes encoding two subunits of glycerol dehydratase reactivating factor, was also digested with restriction enzymes *Aat*II and *Kpn*I, and transferred downstream of the other T7 promotor correspondingly to construct the final expression plasmids pCDFDuet-*pduCDEGH* (C-S302A/Q337A) and pCDFDuet-*pduCDEGH*, respectively. The plasmids were introduced into *E*. *coli* BL21(DE3) and recombinantly expressed by induction with 1mM IPTG at 30°C for 15 h. The cell pellet was washed and resuspended in 20 mM phosphate buffer (pH 8.0), and disrupted with Bead Beater (Mini-Beadbeater-16, BioSpec) for 15s (8 cycles) in an anaerobic condition. The broken cells were centrifuged at 13 500 x g for 30 min at 4°C to remove cell debris, and the supernatant was collected to measure the crude glycerol dehydratase activity.

Construction of recombinant *E*. *coli* strains expressing native PduQ and PduP sequences, respectively, was performed previously [15,27]. Basically, the recombinant *E*. *coli* strains were grown in 500 mL flask containing 100 mL LB medium with 40–100 μg/mL of suitable antibiotics under aerobic conditions at 37°C with shaking at 220 rpm. The gene expression was induced by addition of 0.1–1 mM IPTG when the bacterial growth had reached mid-exponential phase (OD_620_ around 0.5) with subsequent incubation for 24 h at 15°C and 160 rpm. The cells were then harvested and washed with 20 mM sodium phosphate buffer pH 7.4 containing 0.5 M NaCl. Lysis of the cells was performed by BugBuster according to manufacturer´s instructions. The soluble protein fraction was separated from cell debris by centrifugation for 40 min at 16 000 x g, 4°C, and subjected to immobilized metal ion affinity chromatography (IMAC) for purification of the recombinant enzymes. The protein solution was loaded on 5 mL Ni-NTA HisTrap™ FF crude column equilibrated with binding buffer (20 mM sodium phosphate, 0.5 M NaCl, 5 mM imidazole, pH 7.4) at a flow rate of 1 mL/min. After washing the column with the binding buffer, the bound protein was eluted using elution buffer containing 500 mM imidazole solution. Purity of the recombinant enzymes was analysed by sodium dodecyl sulfate-polyacrylamide gel electrophoresis (SDS-PAGE) on a gel containing 12% acrylamide. The protein bands were stained with Coomassie Brilliant Blue R-250.

### Determination of activities of GDH, PduQ and PduP

The activity of crude glycerol dehydratase was determined by the 3-methyl-2-benzothialzolinone hydrazone (MBTH) method as described earlier [28], which is based on the ability of aldehydes formed during the dehydratase reaction to react with MBTH. The resulting azine derivative is detected spectrophotometrically. Generally, the assay mixture (1 mL) contained an appropriate amount of GDH, 0.2 M substrate, 0.05 M KCl, 0.035 M potassium phosphate buffer (pH 8.0), and 15 μM coenyzme B12. After incubation at 37°C for 1–10 min, the enzyme reaction was terminated by adding 1 mL 0.1 M potassium citrate buffer pH 3.6 and 0.5 mL 0.1% MBTH. After 15 min at 37°C, the amount of aldehyde formed was determined by reading the absorbance at 305 nm. One unit of glycerol dehydratase activity is defined as the amount of enzyme activity catalyzing the formation of 1 μmol aldehyde/min at 37°C.

The activity of PduQ was determined as described by Reid et al [29] with some modification. Reduction of the aldehyde substrate was measured by following the decrease in absorbance of reduced nicotinamide adenine dinucleotide (NADH) at a wavelength of 340 nm (∑_340_^NADH^ = 6.22mM^-1^cm^-1^) in a reaction mixture containing 0.2 mM NADH, 100 mM of an aldehyde substrate and appropriate amount of enzyme in 50 mM potassium phosphate buffer pH 7.0. One Unit (U) of activity is defined as the amount of enzyme catalyzing the consumption of 1 μmol NADH per minute.

The activity of PduP was determined by a method developed by Leal et al. [30]. The method is based on the oxidation of the substrate aldehyde in the presence of NAD(P)^+^ and measuring the production of NAD(P)H spectrophotometrically at 340 nm. The reaction mixture containing 0.43 mM HS-CoA and 5 mM NAD^+^ in 50 mM potassium phosphate buffer pH 7.0 supplemented with 1 mM dithiothreitol and 0.1 g/L BSA was equilibrated for 5 min at 30°C before initiating the reaction by addition of 100 mM aldehyde substrate. One Unit (U) of activity is defined as the amount of enzyme catalyzing the formation of 1 μmol NADH formed per minute.

All spectrophotometric measurements were made using UV-1650 PC Spectrophotometer (Shimadzu, Kyoto, Japan).

### Construction of chromosomal *pduC* mutations

Scarless chromosomal site-directed point mutations of *PduC* gene in *L*. *reuteri* were constructed by single-stranded DNA recombineering method as described earlier [31,32], with slight modifications. Briefly, electrocompetent cells, in which RecT is expressed, were transformed with a recombineering oligonucleotide oLCH1(50-mer, 100 μg), which is identical to the lagging strand containing mutiple non-complementary bases. Viable cells were then recovered on antibiotic-free plates and recombinants are detected by a mismatch amplification mutation assay-PCR (MAMA-PCR). Single colony purification was performed to separate the wild-type genotype from the mutant ones. The resultant mutant strain PduC*-*S302A was constructed by curing from pJP042, passing bacteria without antibiotic selection to yield a plasmid-free derivative. A similar strategy was used for constructing double mutant (PduC-S302A/Q337A) by transforming a recombineering oligonucleotide oLCH2 (50-mer, 100 μg) followed by the procedure similar to that described above. All the primers used and plasmids constructed are listed in S1 Table. The *pduC* mutants were also verified by DNA sequencing analysis.

### Biotransformation using resting *L*. *reuteri* cells

*L*. *reuteri* cells were grown in a 3 L bioreactor (Applikon, Microbial Biobundle, The Netherlands) as reported previously [4]. Monitoring and control of all the parameters was done through ez-control unit. Stirrer speed was maintained at 200 rpm, temperature at 37°C and pH at 5.5 by addition of 5 N NH_4_OH. Anaerobic conditions were maintained through continuous bubbling of nitrogen gas. Twenty milliliters of freshly prepared inoculum was aseptically added to 2 L fermentation medium containing 55 g/L MRS broth and 20 mM glycerol, cultivation was continued for 12 h after which the broth was collected and centrifuged at 15 000 x g and 4°C for 5 min. The supernatant was discarded and the cell pellet was used for biotransformation experiments.

Biotransformation of different substrates (i.e. ethane-1,2-diol, glycerol, 1,2-propanediol, 1,2-butanediol, 1,2-pentanediol and 1,2-hexanediol) was done in 50 mL centrifuge tubes. The process was initiated by resuspending the harvested *L*. *reuteri* cells from the previous step in 50 mL solution containing 100–150 mM 1,2-diols/ glycerol substrates to a final density of about 10 g _CDW_/L, mixed by vortexing and incubated in a shaker incubator up to 3 hours at 30°C. One milliliter samples were collected at 30 min intervals, centrifuged and the supernatant was analyzed for the concentration of residual substrate and metabolites formed.

### Bioconversion of 1,2-diols and glycerol by whole cells of recombinant *E*. *coli*

Shake flask experiments were performed using LB Lennox (supplemented with 1.2-diols) medium with modification to the method described previously [33]. One milliliter of overnight culture of the transformants harboring constructed plasmid pCDFDuet:*pduCDEGH* or pCDFDuet:Δ*pduCDEGH*(C-S302A/Q337A) were grown in LB (37°C, 220 rpm) as the pre-inoculum, and 500 μL was added to 100 mL flask consisting of 20 mL LB supplemented with 50 mM 1,2-diols or glycerol, along with appropriate antibiotics (streptomycin, 50 μg/ml) and grown for additional 4–6 hours (37°C, 220 rpm). Following this, 10 μM coenzyme B12 and 0.5 mM IPTG were added to the cultures, and cultivation continued for another 24–36 h at 30°C, 200 rpm. One milliliter samples were withdrawn for product analyses.

### Analyses of substrates and products

Ethane-1,2-diol, glycerol, 1,2-propanediol, 1,2-butanediol, 1,2-hexanediol, acetaldehyde, propionaldehyde, butyraldehyde, pentanaldehyde, hexanaldehyde, ethanol, 1,3-propanediol, 1-propanol, 1-butanol, 1-pentanol, 1-hexanol, acetic acid, 3-hydroxypropionic acid, propionic acid, butyric acid, pentanoic acid and hexanoic acid were measured via HPLC (Jasco, Tokyo, Japan). For HPLC, an Aminex HPX-87H chromatographic column was used with an upstream pre-column (Biorad, Richmond, CA, USA) and a refractive index detector (Perkin Elmer, Series 200a, Waltham, Massachusetts, USA) and an intelligent autosampler (Jasco, Tokyo, Japan). Temperature of the column was maintained at 65°C by a column oven (Shimadzu, Tokyo, Japan). Samples from the bioreactor were diluted with MilliQ water and mixed with 20% v/v sulfuric acid and injected into 0.5 mM sulfuric acid mobile phase.

For quantification of 3-HPA, the modified photometric method according to Circle et al. [34], with acrolein as standard was used. Briefly, 200 μL of the sample (diluted to fit in the range of the assay) was mixed with 150 μL of DL-tryprophan, followed by addition of 600 μL of hydrochloric acid (37%), and incubating the mixture for 20 min at 37°C. The resulting purple colour was measured spectrophotometrically at 560 nm and the absorbance was compared with the standard curve.

## Results

### Comparative genome analysis reveals a diversity of gene cluster for 1,2-diol/ glycerol metabolism

**Fig 2:**
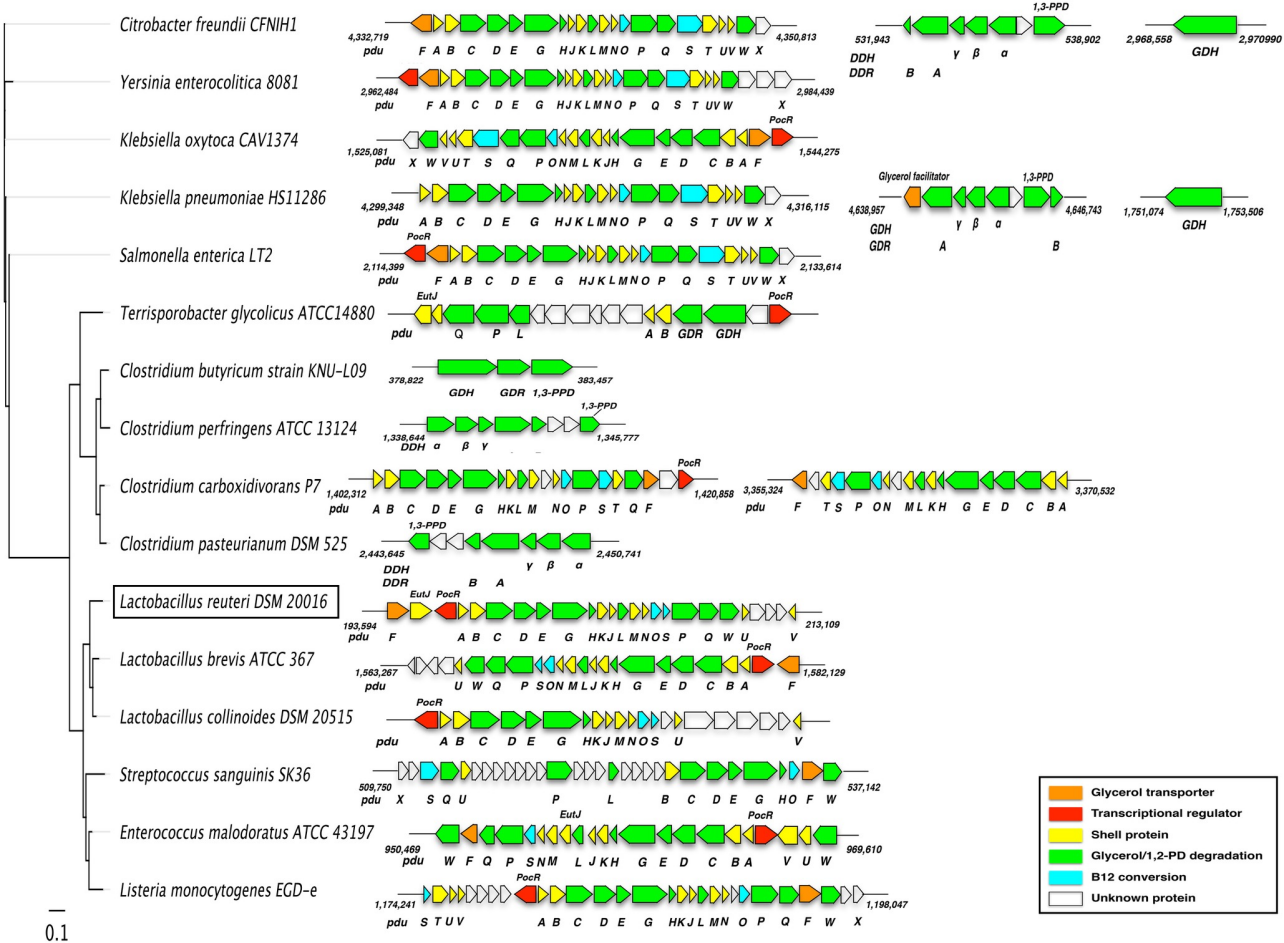
Comparison of the genomic location that contains the pdu gene cluster. Genes in the *pdu* gene cluster are depicted by arrows indicating the transcription direction with the same color codes as decribed in the legend. Phylogenetic relationships were analyzed between the genomes of sequenced strains, including highlighted *Lactobacillus reuteri*, inferred from 16s rRNA amino acid sequences, and an unrooted tree was generated using Geneious. An evolutionary distance was represented by scale bar. Sequences were aligned with ClustalW with bootstrap trial of 100 and bootstrap values (%) are omitted from figure.

Comparison of the genetic loci bearing the pdu gene cluster in different bacteria such as *Citrobacter freundii*, *Salmonella typhimurium*, *Listeria monocytogenes*, *Klebsiella* spp., *Clostridium* spp. and *Lactobacillus* spp. have revealed that the *pdu* genes are clustered in different orders and are scattered differently in the genomes (Fig 2). A detailed comparison performed in the present work, revealed that the *pdu* operon in most of the bacteria listed, except for *C*. *butyricum*, *C*. *perfringens* and *C*. *pasteurianum* that lack the Pdu pathway and the microcompartment, ranges from 15 208 bp (*C*. *carboxidivorans* P7) to 27 392 bp (*S*. *sanguinis* SK36). Diol/glycerol dehydratase, is the key and rate-limiting enzyme as reported previously [35]. The general features of all diol/glycerol dehydratase gene sequences and the gene products are summarized in S2 Table. Particularly, the genes encoding this enzyme found in *C*. *freundii*, *Y*. *enterocolitica*, *K*. *oxytoca*, etc., consist of three types of subunits, i.e, *pduC* (α), *pduD* (β) and *pduE* (γ). Reactivating factors for diol/glycerol dehydratase were also discovered [36,37]. The *gdrA* and *gdrB*, two open-reading frames of *C*. *freundii*, are highly homologous to the products of *gdrA* and *gdrB* of *K*. *pneumoniae* (KPHS_46160, KPHS_46220), *C*. *perfringens* (CPF_RS05765, CPF_RS05770) and *C*. *pasteurianum* (CLPA_RS11070, CLPA_RS11065). Moreover, the *pduG* and *pduH* gene products, which are homologous among all bacteria harboring Pdu-microcompartment, except for *Terrisporobacter glycolicus*, are considered to be involved in the reactivation of glycerol-inactivated diol/glycerol dehydratase. Some bacteria, including *L*. *reuteri*, possess the *pdu-cbi-cob-hem* cluster comprising 58 ORFs, encoding the biosynthetic machinery for the Vitamin B12 cofactor (data not shown). The different arrangements of the diol/ glycerol dehydratase genes offer us alternative options to choose engineering method for modifying the enzyme features and to solve problems of coenzyme inactivation.

*L*. *reuteri* contains three genes (Lreu_1747–1745) with dehydratase subunit motifs in the propanediol untilizing operon (193594–213109 bp). The encoded proteins of these three genes display sequence similarity to PduCDE of other *Lactobacillus* spp. (i.e. *L*. *reuteri*, *L*. *brevis*, *L*. *collinoides*), which share a high level of conserved ORFs in the *pdu* operon. The genomes of *L*. *reuteri* DSM 20016 and *L*. *brevis* ATCC 367 each consist of sequenced circular chromosomes of 199 9618 and 229 1220 bp, and totally contain 2049 and 2030 genes, respectively. A comparison of the *pdu* operon in the two lactobacilli also reveals a conserved gene order (i.e. *pduFABCDEGHKJLMNOSPQWUV*). The close phylogenetic relationship is evident from a comparative analysis of the ribosomal protein sequences from these two lactobacilli (Fig 2), which are both probiotics and with a potential in production of various chemicals, e.g. 3-hydroxypropionaldehyde, 1,3-propanediol and 3-hydroxypropionic acid [14]. On the other hand, the *cbi-cob-hem* gene set present in *L*. *reuteri* has not been identified in other lactobacilli including *L*. *brevis* (data not shown).

### Activities of the key Pdu enzymes involved in the anaerobic metabolism of 1,2-diols/glycerol

**Fig 3:**
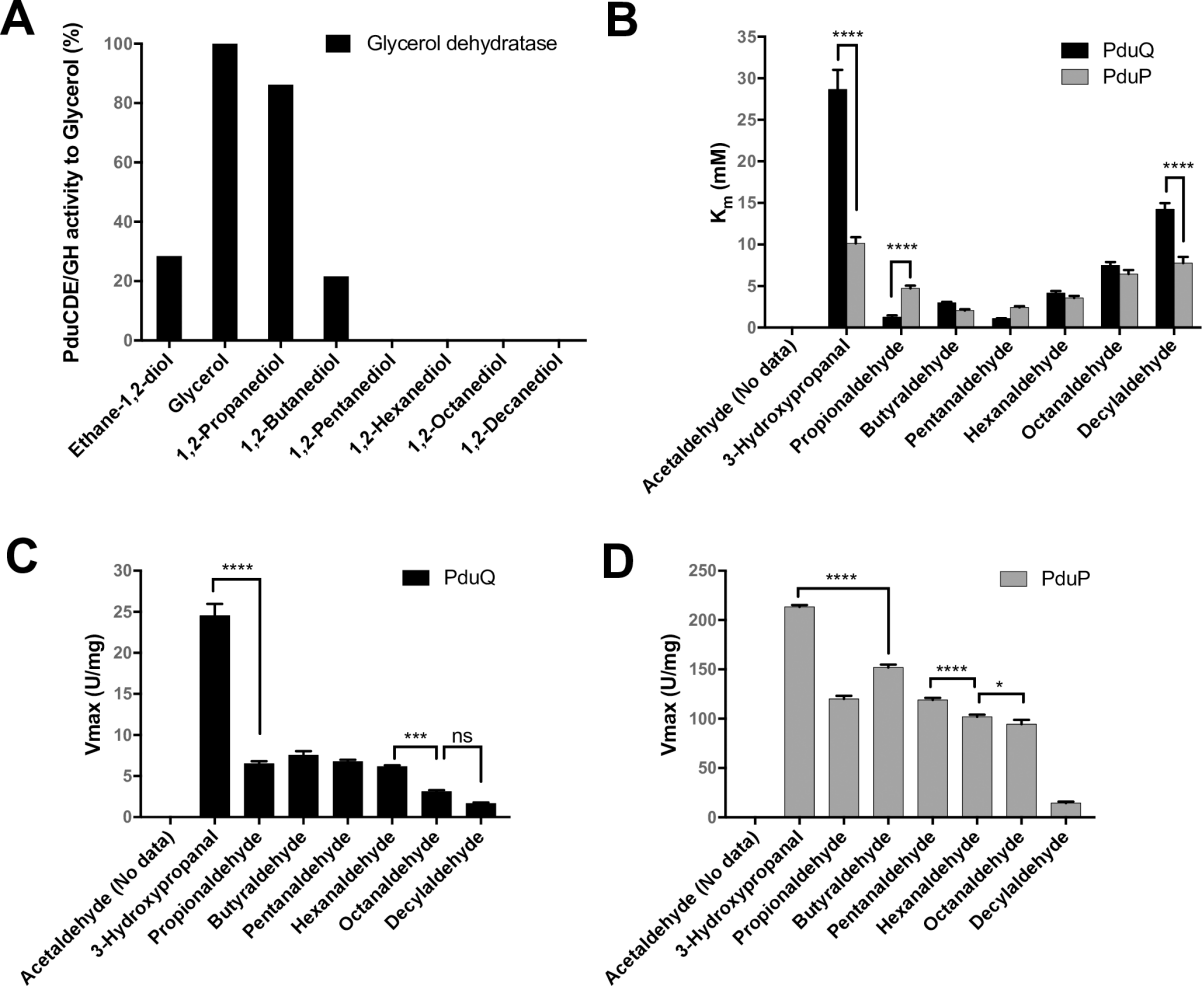
Kinetic analysis of lumen enzymes towards different substrates. A) Relative activities of cell free extracts of recombinant *E*. *coli* expressing wild type glycerol dehydratase toward glycerol/1,2-diols, using the activity towards glycerol as 100%. B) *K*_m_ values for PduQ and PduP towards different aldehydes. Vmax values for C) PduQ and D) PduP.

The three genes *pduCDE* encoding the three subunits of the glycerol dehydratase, together with two genes *pduGH* encoding the two subunits of the glycerol dehydratase activase were cloned in the pCDFDuet-1 expression vector system. Cell-free extracts of *E*. *coli* BL21(DE3) strain show the recombinantly expressed proteins at 62.1 kDa (PduC), 25.8 kDa (PduD), 19.2 kDa (PduE), 65.7 kDa (PduG) and 13.4 kDa (PduH) (S2 Fig). and when tested for dehydratase activity against different 1,2-diols and glycerol, the activity was observed to decrease in the order glycerol> 1,2-propanediol> 1,2-ethanediol> 1,2-butanediol, and no activity was observed with longer chain 1,2-diols (Fig 3A).

Subsequently, activities of the next enzymes in the Pdu pathway, PduQ and PduP were determined against aldehydes of varying chain length. The two enzymes were produced recombinantly and purified prior to activity measurements. As shown in Fig 3B–3D, both the enzymes were active with all aldehydes (C3-C10), except for acetaldehyde (C2) that is extremely volatile. Significantly higher specfic activities was observed for PduP. Highest specific activities for both PduQ and PduP were indeed found with 3-hydroxypropanal (3-HPA) as substrate, with *K*_m_ values of 28.7 mM and 10.1 mM, respectively. With propionaldehyde, PduQ and PduP displayed only 26.5% and 51.9% activities, respectively, compared to that with 3-HPA. Decrease in activity with increasing chain length of the diol substrates seemed to be correlated with the apparent increasing order of *K*_m_ values for both enzymes: Pentanaldehyde (C5)< Hexanaldehyde (C6)< Octanaldehyde (C8)< Decylaldehyde (C10).

### Redesign of glycerol dehydratase to act on longer chain 1,2-diols

**Fig 4:**
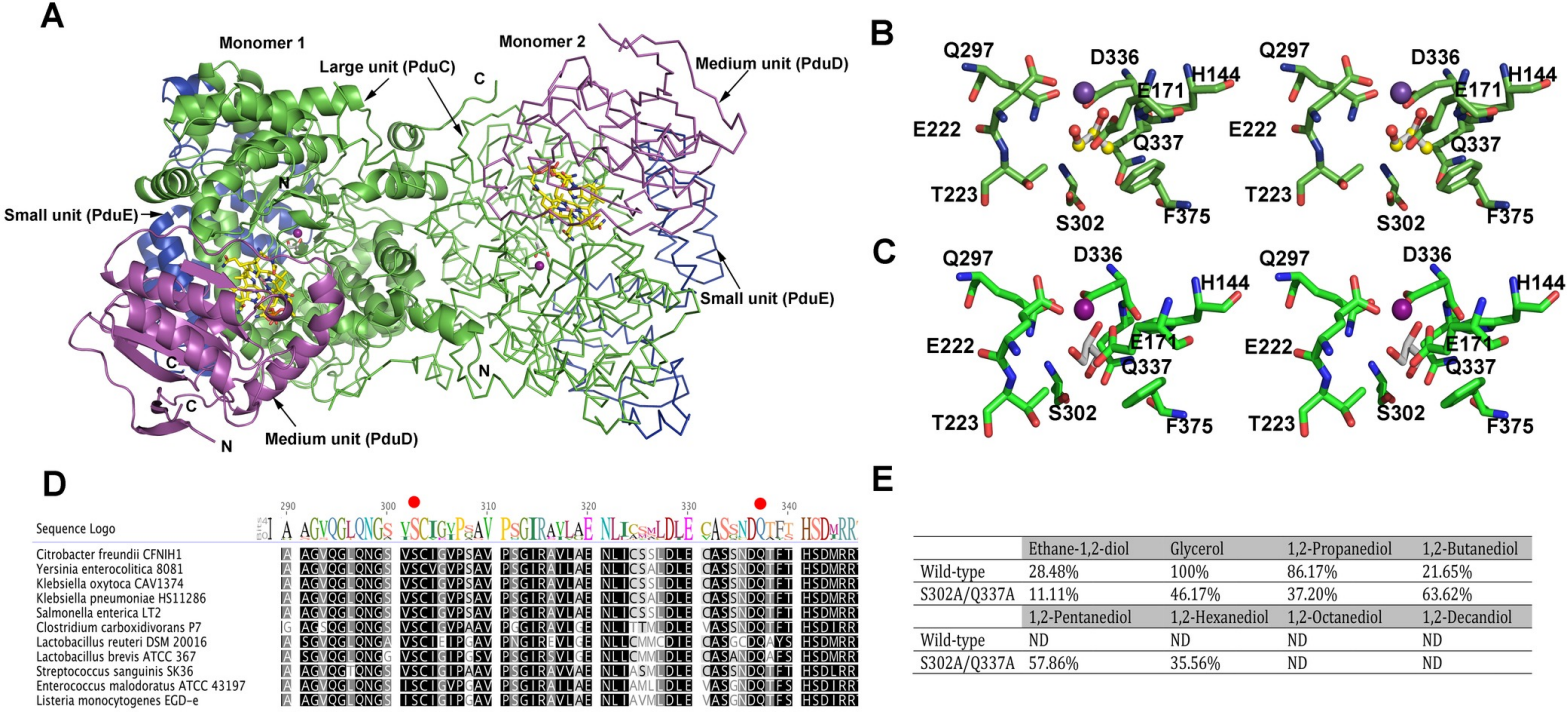
Redesign of glycerol dehydratase from *L*. *reuteri*. A) Overall fold of the glycerol dehydratase monomer and the proposed biological dimer; Stereodrawing of the B) 1,2-PD or C) glycerol bound form of the glycerol dehydratase. Homology modeling for the prediction of tertiary structure of glycerol dehydratase (PduCDE) resulted in a model based on the crystal structure of diol dehydratase-cyanocobalamin complex from *Klebsiella oxytoca* (1DIO), with sequence similarities and coverages above 42% and 95%, respectively. Quality model assessment of the homology model revealed a QMEAN6-score of 0.83 and a Z-score of -0.53. Ramachandran plot revealed that none of residues were present in the disallowed regions. Color codes for carbon atoms: yellow for adenosylcobalamin (AdoCbl), white for glycerol/1,2-PD, purple for calcium ion. The model figure was generated using Pymol. D) Sequence alignment of large subunit of glycerol dehydratase (PduC) from different bacteria. The numbering scheme follows the amino acid sequences of PduCs. Identical residues in all sequences are highlighted in black and conserved in grey. E) Relative activities of WT/mutant glycerol dehydratases towards different 1,2-diols/glycerol. The data is normalized by taking the activity of the wild-type enzyme towards glycerol as 100%.

As the activity of glycerol dehydratase is a limiting step in bioconversion of different 1,2-diols by *L*. *reuteri*, broadening of substrate selectivity of the enzyme by rational engineering could contribute to transformation of longer chain 1,2-diols. Therefore, comparison of the binding model of glycerol and 1,2-propanediol to glycerol dehydratase was first drawn based on homology modeling (Fig 4). The model based on the crystal structure of diol dehydratase-cyanocobalamin complex from *Klebsiella oxytoca* (1DIO) [38], revealed sequence similarity of 42% and coverage above 95%. As shown from the model, glycerol dehydratase is assembled in the form of a dimer of PduCDE heterotrimers. The large subunit (PduC), harboring an essential cofactor ion-binding site, contains a TIM barrel structure with the active site isolated inside the central barrel formed by eight paralled β strands. Coenzyme B_12_ is located in between the PduC and medium subunit (PduD). It was also observed from the docking model that on one hand, 3-OH group of glycerol forms a hydrogen bond with the side chain of S302 while on the other, the side-chain amide nitrogen atom of Q337 formed hydrogen bonds with the main chain carbonyl oxygen atoms of D336 and F375, both of which are key residues for interacting with substrate molecule (S1 Fig). Loss of these hydrogen bonds may alter the conformation and the flexibility, therefore decreasing the bulkiness of the residues in these positions.

Hence, according to the hypothesis that a less bulky residue could yield more space for accomodating a longer alkyl group on C3 of 1,2-diols, the plasmid for the mutant glycerol dehydratase was constructed in which both of the residues (S302 and Q337) were substituted by alanine. The relative activities of cell-free extract of the wild-type and the mutant enzymes towards different 1,2-diols and glycerol were compared. As shown in Fig 4E, the S302A/Q337A mutation resulted in decrease in relative activity towards 1,2-ethanediol, glycerol and 1,2-propanediol to 39.0%, 46.2% and 43.2%, respectively, of the wild type. In contrast, relative activity towards 1,2-butanediol increased by almost three folds. Moreover, activities towards longer chain 1,2-diols, i.e. 1,2-pentanediol and 1,2-hexanediol, were also detected with relative increase by 57.86% and 35.56%, respectively.

### Whole cell bio-transformation of 1,2-diols/glycerol by engineered *L*. *reuteri*

**Fig 5:**
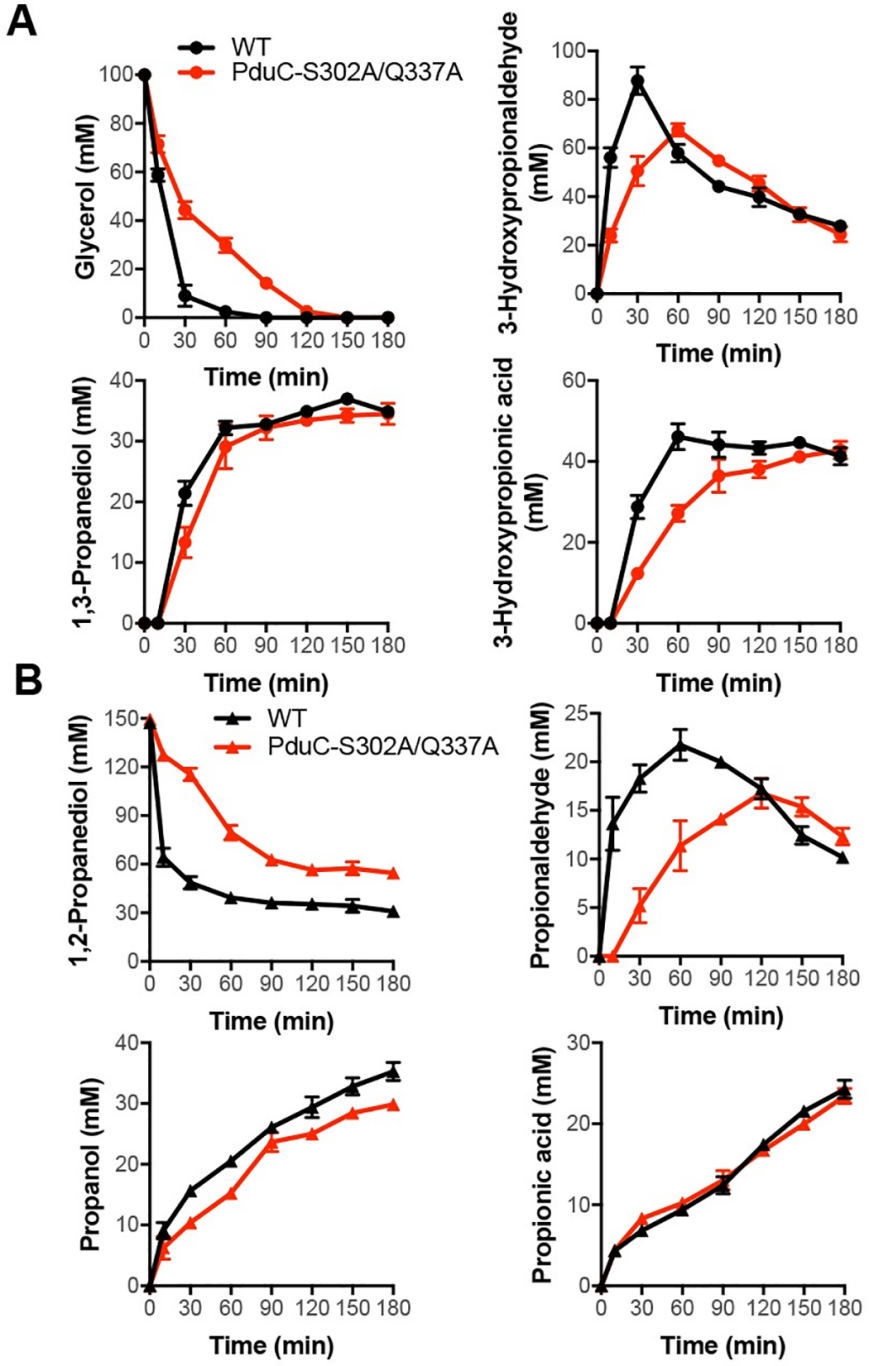
Time course of biotransformation of glycerol and 1,2-PDO using wild-type and mutant *L*. *reuteri*.

In order to study the effect of the mutation in glycerol dehydratase described above on the enzyme activity on different 1,2-diols in whole cells, *L*. *reuteri* was engineered to create a glycerol dehydratase variant (S302A/Q337A). As seen in Fig 5, no detectable quantities of products were observed from cultures fed with 1,2-ethanediol, 1,2-butanediol, 1,2-pentanediol and 1,2-hexanediol. Furthermore, it was seen that the S302A/Q337A mutant cells showed a negligible difference in resistance toward substrate inhibition by glycerol. The native cells (11.3 g _CDW_/L) converted 6 mmoles 1,2-PD within 3 h, while the mutant cells (10.8 g _CDW_/L) showed a 20% decrease in conversion efficiency (4.8 mmoles), leading to *n*-propanol production at 1.45 mmoles, as compared to 1.75 mmoles from native cells (Fig 5).

### Recombinant *E*. *coli* cells bearing the glycerol dehydratase variant transform longer chain 1,2-diols

**Fig 6:**
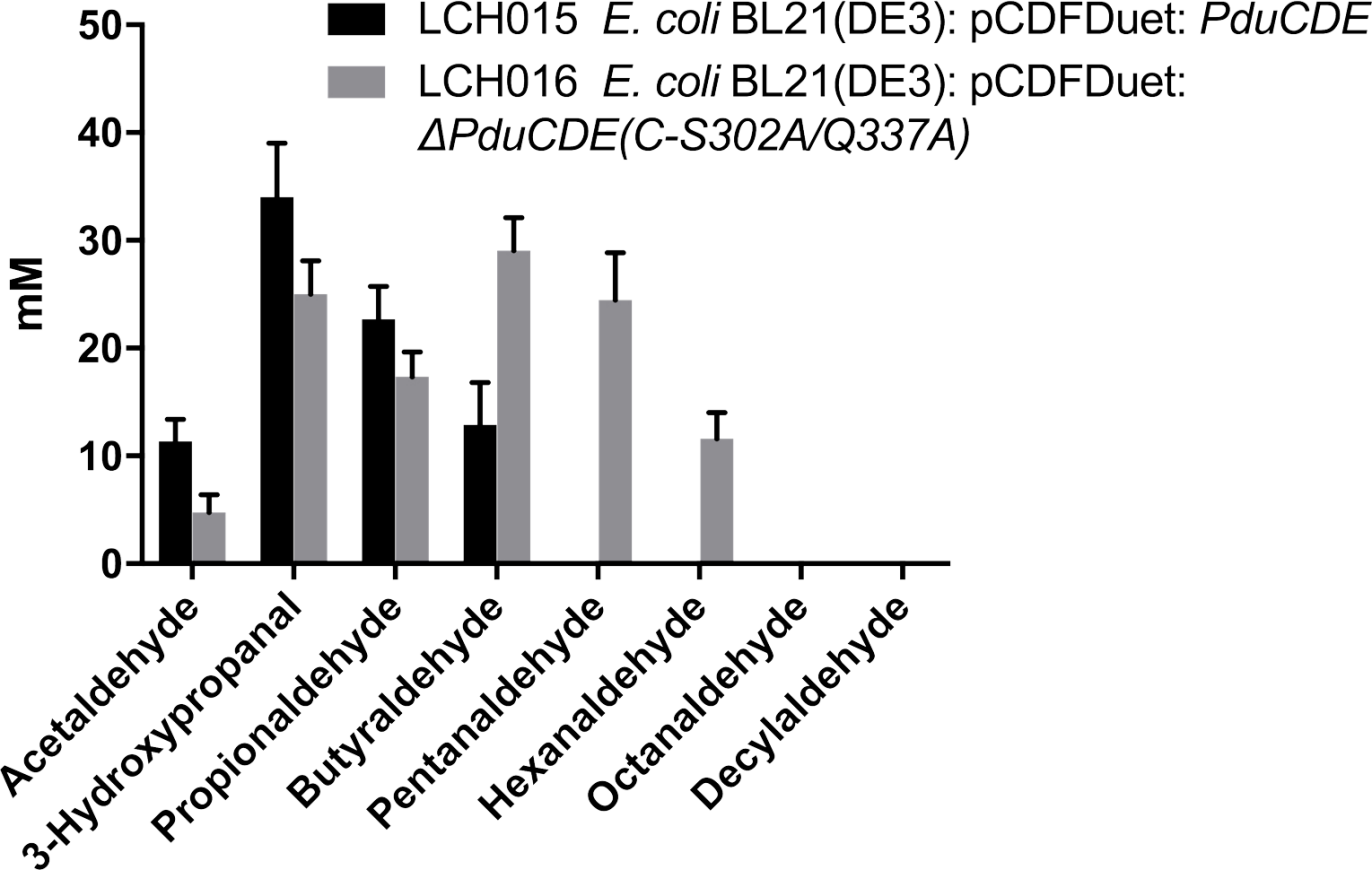
Results of shake flask studies (36 h) for *de novo* conversion of 1,2-diols using resting cells of recombinant *E*. *coli* strains.

In order to confirm that the lack of activity of the engineered whole cells of *L*. *reuteri* towards the longer substrates was due to the diffusional barrier posed by the microcompartment, the reactions with the 1,2-diols were tested with recombinant *E*. *coli* cells in which the glycerol dehydratase with the native and mutated sequence, respectively, were recombinantly expressed. In contrast to the cells with glycerol dehydratase having the native amino acid sequence that showed highest activity with glycerol and no activity beyond 1,2-butanediol, the cells bearing the enzyme variant LCH016 showed activity with substrate chain length up to C6 and the highest activity with C4. The product titers achieved from reaction with 2.5 mmoles substrates using by 0.98 g _CDW_/L cells were 0.24± 0.08 mmoles acetaldehyde, 1.25± 0.16 mmoles 3-hydroxypropanal, 0.87± 0.12 mmoles propionaldehyde, 1.46± 0.16 mmoles butyraldehyde, 1.23± 0.22 mmoles pentanaldehyde and 0.58± 0.12 mmoles hexanaldehyde, respectively (Fig 6).

## Discussion

The biotechnological transformation of 1,2-propanediol/glycerol to alcohol and acid by the Pdu pathway has been demonstrated in several bacteria such as *L*. *brevis*, *L*. *reuteri*, *L*. *buchneri*, *C*. *freundii*, *K*. *pneumoniae* and *C*. *butyricum* [13,39–42]. Glycerol bioconversion to 1,3-propanediol has however attracted greater interest from an industrial viewpoint [43–45]. Comparative genome analysis has shown these bacteria to possess a specific gene cluster encoding the pathway enzymes and the protein shell forming the microcompartment, providing an optimum environment for efficient transformation and minimising the inhibitory effects of the intermediates and products.

Expanding the repertoire of diol substrates for transformation by the Pdu pathway could result in a novel synthetic route for the production of interesting products. However, establishing routes for microbial production of chemicals often requires overcoming pathway bottlenecks by tailoring enzymes to accept non-native substrates. Diols are important chemicals with applications as building blocks for polymers, in cosmetics and pharmaceuticals, and as fuels [46]. 1,2-Diols, namely 1,2-propanediol, 1,2-butanediol, 1,2-pentanediol, 1,2-hexanediol can be biotechnologically produced by direct microbial bioconversion of renewable feedstocks [47].

Diol/glycerol dehydratases have been recognised for their catalytic ability with C2 (1,2-ethanediol) and C3 (1,2-propanediol) diol, and C3 (glycerol) [48–50]. In this study, we found that the relative substrate activities towards 1,2-PDO was 86% with respect to glycerol, which is consistent with the previous studies [51] suggesting the wide-type dehydratase of *L*. *reuteri* has a higher affinity for glycerol than for 1,2-propanediol (*K*_*m*_ 3.3 mM vs 7 mM). Earlier studies, including those from our laboratory, have further shown that *L*. *reuteri* is unique among the lactobacilli in its ability to accumulate and excrete 3-HPA, and is also tolerant to relatively high concentration of the aldehyde product [14,51]. Reactions with other 1,2-diols showed that the wild-type enzyme showed activity with 1,2-ethanediol and 1,2-butanediol only, corresponding to 28% and 21% activity relative to glycerol. Lack of activity with longer chain diols is attributed to the less space available for accomodating a longer alkyl chain in the active site [52].

The active subunit PduC of diol/glycerol dehydratase is quite conserved among the bacteria (Fig 4D). It was foreseen by docking analysis that replacing S302 and Q337 by Alanine residues would result in the loss of hydrogen bonds between the active site and substrate, altering the flexibility of the binding site. Activity measurements of the glycerol dehydratase with double mutations showed the increased substrate scope of the the enzyme variant. It is especially evident that the relative acitivity towards ethane-1,2-diol, glycerol and 1,2-propanediol decreases significantly, to 11%, 46% and 37%, respectively, with respect to the 100% activity of the wild type enzyme with glycerol (Fig 4E), most likely due to the reduced contact between the substrates and the side chains of the mutated residues in glycerol dehydratase. These results are in close agreement with the observations made by Yamanishi and coworkers, who mutated the same residues in *Klebsiella oxytoca* diol dehydratase to increase the resistance of the enzyme towards mechanism-based inactivation by glycerol [52]. They reported increased *K*_*m*_ value of the mutants towards 1,2-PDO and increased activity towards longer chain 1,2-diols as a result of the reduced bulkiness of the residues in the catalytic position. Unlike the *K*. *oxytoca* enzyme however, the *L*. *reuteri* GDH has shown negligible difference in resistance towards inhibition by glycerol (unpublished data), confirming the previous assumption that *L*. *reuteri* enzyme is a glycerol dehydratase that is more resistant towards substrate inhibition.

Interestingly, unlike the GDH both PduP and PduQ were able to transform aldehydes of chain lengths up to C10, although PduQ exhibited lower specific activity that could partly be ascribed to metal induced loss of activity on exposure to air (unpublished observations). Nevertheless, this result implies that the combined use of the PduP and PduQ branches of the Pdu pathway would provide a unique biotransformation system for transformation of aldehydes simultaneously to an acid and an alcohol product, a biocatalytic alternative to the chemical Canizzaro reaction involving base induced disproportionation of an aldehyde.

Since *L*. *reuteri* possesses the entire Pdu pathway including the machinery for activating the cofactor needed for glycerol dehydratase, and has been shown earlier to efficiently transform glycerol to 3-HPA, 3-HP and 1,3-PDO with relatively high tolerance to product inhibition [4,9,10], expanding the repertoire of diol substrate for transformation by the microorganism would result in a novel biosynthetic system for specific aldehyde, acid and alcohol products. However, inspite of the broader substrate scope of the mutant glycerol dehydratase and also of PduP and PduQ, diols longer than 1,2-PDO could not be transformed by the whole cells of *L*. *reuteri* cells. This was ascribed to the barrier posed by the protein shell of microcompartment for the passage of the more longer, hydrophobic diols. That this was really the case was supported by the ability of recombinant *E*. *coli* expressing the GDH variant to transform 1,2-diols up to C6 to their corresponding aldehydes in accordance with the substrate selectivity of the double mutant. Notwithstanding, the disadvantage of using recombinant *E*. *coli* (e.g. BL21(DE3) and its derivatives) is obvious, even though cells harboring engineered glycerol dehydratase can convert certain amount of long chain diols. One of the most important points is that the ability of *E*. *coli* to survive in low pH condition is much weaker as compared to *L*. *reuteri*, which can be a promising host for low-pH fermentation.

Crowley et al. identifed that the properties of the PduA pore of *Salmonella enterica* is suggestive of 1,2-PDO transport [53]. Structural analysis of the shell protein PduB of *Lactobacillus reuteri* revealed that besides glycerol occupying the subunit channels of PduB, the trapped glycerol in a central pocket locks the loops closed and raises the possibility of a ligand-gated channel [54]. From our previous work on *L*. *reuteri* DSM20016, it is well established that the Pdu enzymes (PduCDE, PduP, PduL, PduQ etc.) are packed within the microcompartment of *L*. *reuteri* and the shell is permeable to 1,2-propanediol/glycerol (substrates), 3-hydroxypropionaldehyde (3-HPA), propanol/1,3-propanediol and propionyl-phosphate/3-hydroxypropionyl-phosphate, but propionaledhyde is somehow sequestered to prevent cell damage [15]. There are some hints that limit of microcompartment may be caused by polarity of the pore site that can affect transport of both small molecules (e.g. 3-HPA and propionaldehyde) and large cofactors (e.g. HS-CoA and NAD^+^/NADH) [55–58]. However, the underlying mechanism is still not entirely clear and is being investigated by us. In our ongoing work on mutations of *L*. *reuteri* shell protein PduA/J pore amino acid residues, certain mutants perform differently than the wild type (unpublished data), which also indicates that the physicochemical properties of the Pdu-microcompartment pore site could determine the interaction and diffusion of 1,2-propanediol/glycerol or longer chain 1,2-diols through the pores.

While the present studies have involved coenzyme B_12_-dependent diol/glycerol dehydratase, the research could be further extended to B_12_-independent enzymes, e.g. from *C*. *freundii* CFNIH1, *K*. *pneumoniae* HS11286, *C*. *butyricum* KNU-L09 and *T*. *glycolicus* ATCC14880 (S2 Table) due to their less complexity and low cost for reactivation for large scale applications. Improvements in biotransformations can further be achieved by using a more active PduQ or a similar enzyme, and by replacing the oxidative branch of the Pdu pathway with a single step of aldehyde oxidation using a suitable aldehyde dehydrogenase. *L*. *reuteri* does indeed possess a cytoplasmic alcohol dehydrogenase [15] and an aldehyde dehydrogenase with activity against 3-HPA, which could be evaluated for their action on longer chain substrates.

## Supporting information

S1 Fig Sequence and struture analysis of glycerol dehydratase (PduCDE). (TIF)

S2 Fig Agarose gel analysis of DNA sequences of glycerol dehydratase (PduCDE/DhaB) (lane b), glycerol dehydratase reactivating factor (PduGH/GdrAB) (lane c), DhaBgdrA (lane d) and gdrB (lane e); and SDS-PAGE analysis on 12% acrylamide gel of BL21(DE3):pETCDuet cell lysate (4 μg, lane g), and BL21(DE3):pETCDuet:*pduCDEGH* cell lysis (6.5 μg, lane h). Standard nucleotide and protein ladder are shown in lanes a and f.
(TIF)

S3 Fig SDS-PAGE analysis of purification of (A) PduQ and (B) PduP using 12% acrylamide gel. BL21(DE3):pET21a:*pduQ* and BL21(DE3):pET28b:*pduP* cell lysate are shown in lane b, respectively, while the purified PduQ (15 μg) and PduP (10 μg) are in lane c, respectively. Standard protein ladder is shown in lane a.
(TIF)

S1 Table Strains, plasmids and primers used in the study. (DOCX)

S2 Table Properties of the genes and the corresponding gene products for the three structural subunits of glycerol dehydratase. (DOCX)
