# Supplementary figures and images for: Exploring Lactobacillus reuteri DSM20016 as a biocatalyst for transformation of longer chain 1,2-diols: Limits with microcompartment
Source: PLoS One. 2017 Sep 28;12(9):e0185734. doi: 10.1371/journal.pone.0185734 (PMC5619818; doi:10.1371/journal.pone.0185734)

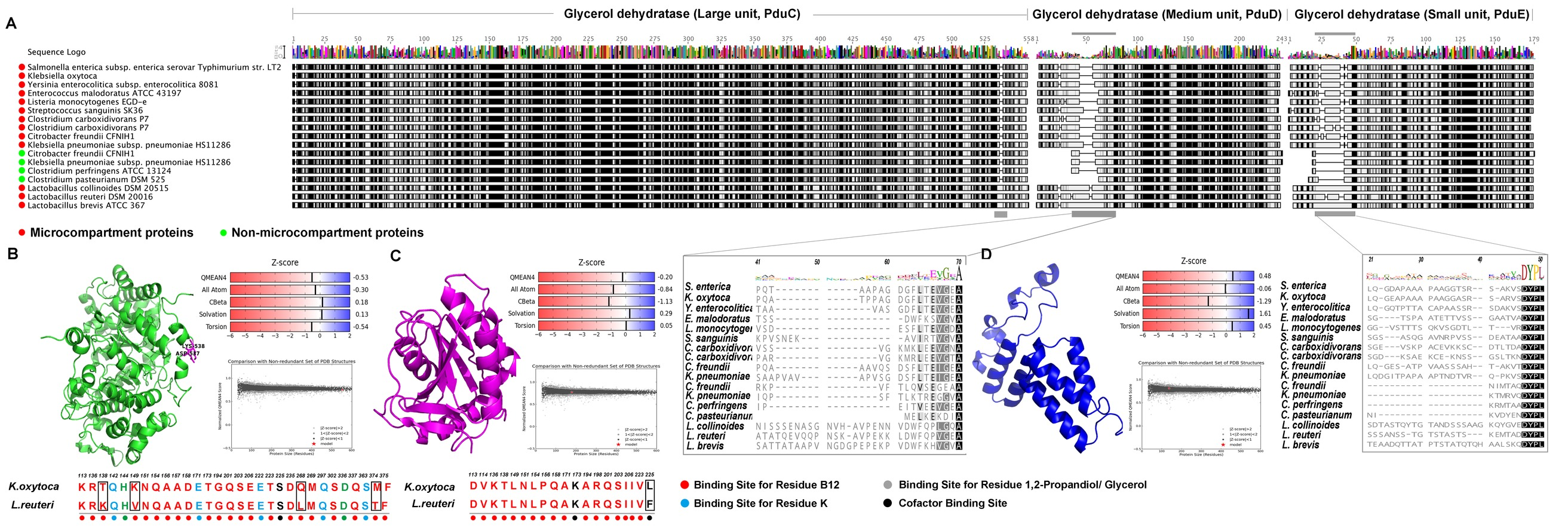

Supplement: S1 Fig — (TIF) [file pone.0185734.s001.tif]

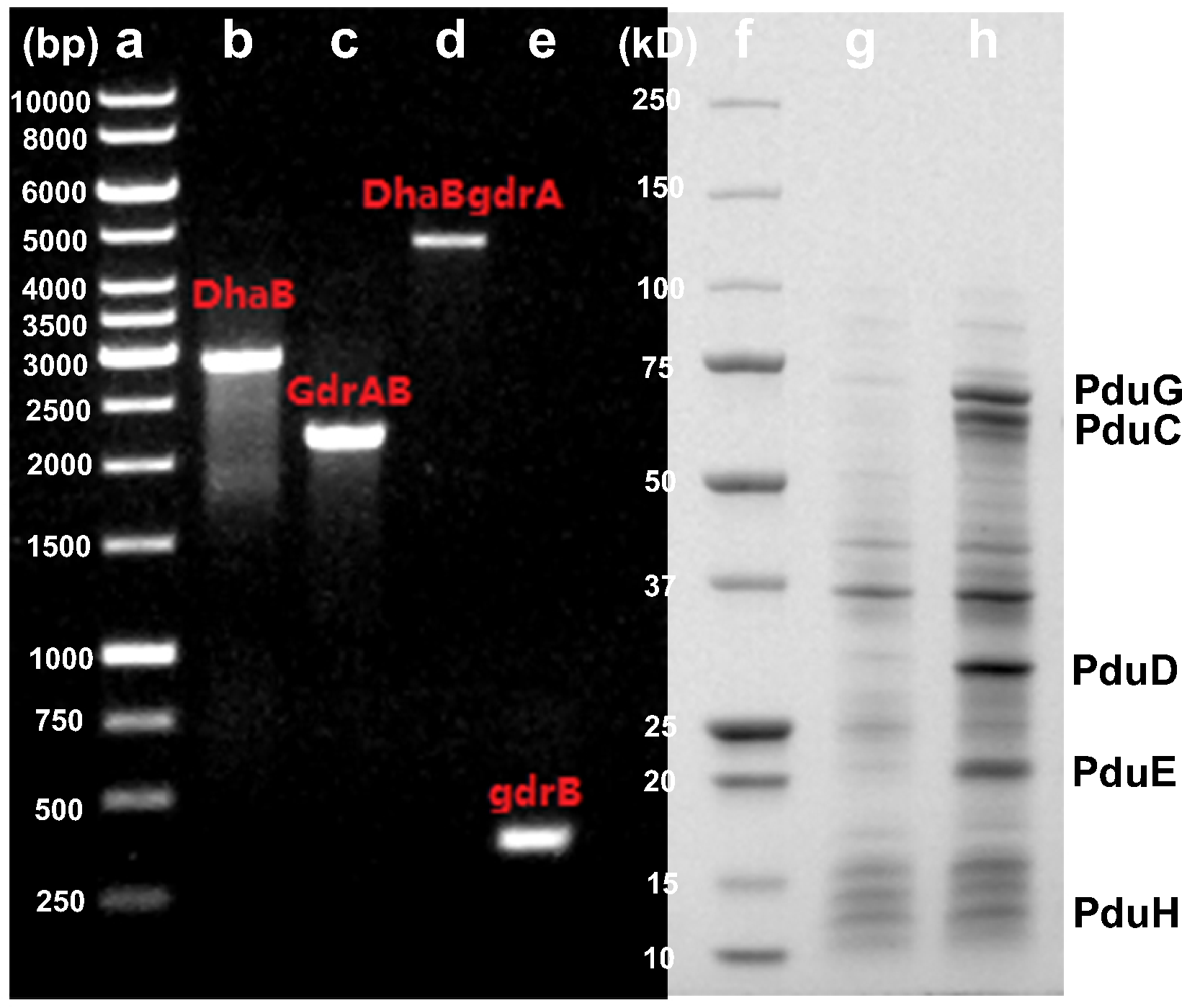

Supplement: S2 Fig — Agarose gel analysis of DNA sequences of glycerol dehydratase (PduCDE/DhaB) (lane b), glycerol dehydratase reactivating factor (PduGH/GdrAB) (lane c), DhaBgdrA (lane d) and gdrB (lane e); and SDS-PAGE analysis on 12% acrylamide gel of BL21(DE3):pETCDuet cell lysate (4 μg, lane g), and BL21(DE3):pETCDuet:pduCDEGH cell lysis (6.5 μg, lane h). Standard nucleotide and protein ladder are shown in lanes a and f. (TIF) [file pone.0185734.s002.tif]

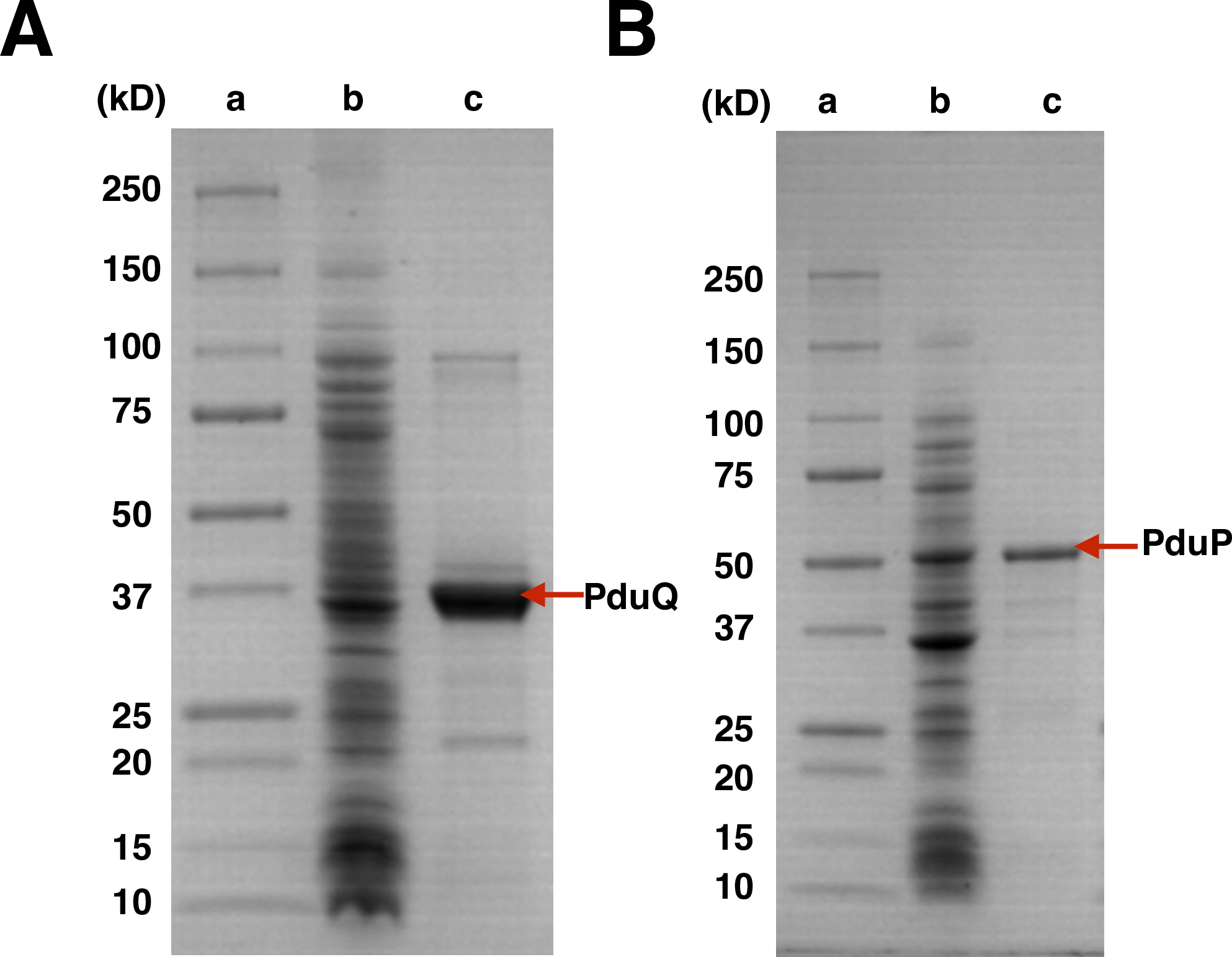

Supplement: S3 Fig — SDS-PAGE analysis of purification of (A) PduQ and (B) PduP using 12% acrylamide gel. BL21(DE3):pET21a:pduQ and BL21(DE3):pET28b:pduP cell lysate are shown in lane b, respectively, while the purified PduQ (15 μg) and PduP (10 μg) are in lane c, respectively. Standard protein ladder is shown in lane a. (TIF) [file pone.0185734.s003.tif]
