# Supplementary material for: Exploring Lactobacillus reuteri DSM20016 as a biocatalyst for transformation of longer chain 1,2-diols: Limits with microcompartment
Source: PLoS One. 2017 Sep 28;12(9):e0185734. doi: 10.1371/journal.pone.0185734 (PMC5619818; doi:10.1371/journal.pone.0185734)
Supplement: S1 Table — (DOCX) [file pone.0185734.s004.docx]

|  | **Material** | **Description** | **Source** |
| --- | --- | --- | --- |
| **Strains** | *E. coli* XL1-blue | Cloning host | Novagen |
|  | *E. coli* DH5α | Cloning host | Novagen |
|  | *E. coli JM109* | Cloning host | Novagen |
|  | *E. coli* BL21(DE3) | Expression host | Novagen |
|  | *L. reuteri* DSM 20016 | WT strain; Source of *pduC*, *pduD*, *pduE*, *pduG*, *pduH*, *pduP* and *pduQ* gene | DSMZ |
|  | LCH013 | Derivative of wide-type *L.reuteri*, *pduC::*oLCH1 (S302A) | This study |
|  | LCH014 | Derivative of LCH013, *pduC::*oLCH2 (S302A, Q337A) | This study |
|  | LCH015 | Derivative of *E. coli* BL21(DE3), *E. coli* BL21(DE3): pCDFDuet: *PduCDE* | This study |
|  | LCH016 | Derivative of LCH015, *E. coli* BL21(DE3): pCDFDuet:  *ΔPduCDE(C-S302A/Q337A)* | This study |
| Plasmids |  |  |  |
|  | pGEM-T | *lac*Zα; cloning vector; pGEM 5zf(+) derivative; 3’T-overhang; Amp^R^ | Promega |
|  | pCDFDuet-1 | *Lac*I; expression vector; T7 promoter; CloDF13-ori; two sets of MCS; MCS I, His_6_-N; MCS II, S-tag-N; Sm^R^ | Novagen |
|  | pCOLADuet-1 | Expression vector, Kn^R^ | Novagen |
|  | pET21a | Expression vector, Amp^R^ | Novagen |
|  | pET28b | Expression vector, Kn^R^ | Novagen |
|  | pET28b: *pduP* | pET28b vector with *pduP*, Kn^R^ | Lab collection |
|  | pET21a: *pduQ* | pET21a vector with *pduQ*, Amp^R^ | Lab collection |
|  | pGEM-T: *pduCDE* | pGEM-T vector with *pduCDE*, Amp^R^ | This study |
|  | pGEM-T: *pduGH* | pGEM-T vector with *pduGH*, Amp^R^ | This study |
|  | pGEM-T: *pduCDEGH* | pGEM-T vector with *pduCDEGH*, Amp^R^ | This study |
|  | pGEM-T: *pduCDE* (C-S302A) | pGEM-T vector with *pduCDE* single site mutant (C-S302A), Amp^R^ | This study |
|  | pGEM-T: *pduCDE*  (C-S302A/ Q337A) | pGEM-T vector with *pduCDE* double sites mutant (C-S302A/Q337A), Amp^R^ | This study |
|  | pCDFDuet: *pduCDE* | pCDFDuet vector with *pduCDE*, Sm^R^ | This study |
|  | pCDFDuet: *pduCDE*  (C- S302A/ Q337A) | pCDFDuet vector with *pduCDE* double sites mutant (C-S302A/Q337A), Sm^R^ | This study |
|  | pCDFDuet: *pduGH* | pCDFDuet vector with *pduGH*, Sm^R^ | This study |
|  | pCDFDuet: *pduCDEGH* | pCDFDuet vector with *pduCDEGH*, Sm^R^ | This study |
|  | pCDFDuet: *pduCDEGH*  (C- S302A/Q337A) | pCDFDuet vector with *pduCDEGH* double sites mutant (C-S302A/Q337A), Sm^R^ | This study |
|  | pSIP411 | EmR, Sakacin-P based expression vector | Lab collection |
|  | pJP042 | Derivative of pSIP411, replaced *gusA* with *recT1* | Lab collection |
| Primers |  |  |  |
|  | prLCH-PduCDE_F_BamHI | CT**GGATCC**GATGAAACGTCAAAAACGATTT | This study |
|  | prLCH-PduCDE_R_EcoRI | CC**GAATTC**TTAGTTATCGCCCTTTAGCTTCT | This study |
|  | prLCH-PduGH_F_AatII | TGGTA**GACGTC**ATGGCAACTGAAAAAGTAATTG | This study |
|  | prLCH-PduGH_R_KpnI | GTT**GGTACC**TCACCTGTTTGCCATTTCCTT | This study |
|  | prLCH-PduCDEG_F_BamHI | CT**GGATCC**GATGAAACGTCAAAAACGATTT | This study |
|  | prLCH-PduCDEG_R_SacI | G**GAGCTC**TCATCGTTGTTCATAACGCTTC | This study |
|  | prLCH-PduH_F_SacI | T**GAGCTC**GATGAACAACGATGATTCACAAC | This study |
|  | prLCH-PduH_R_AflII | GGCT**CTTAAG**TCACCTGTTTGCCATTTCCTTA | This study |
|  | prLCH-PduC-S302A-F | CAAAATGGTGCCGTA**GCC**TGTATTGAAATTCCT | This study |
|  | prLCH-PduC-S302A-R | AGGAATTTCAATACA**GGC**TACGGCACCATTTTG | This study |
|  | prLCH-PduC-Q337A-F | GCTTCTGGTTGTGAC**GCA**GCATACTCACACTCC | This study |
|  | prLCH-PduC-Q337A-R | GGAGTGTGAGTATGC**TGC**GTCACAACCAGAAGC | This study |
|  | prLCH-PduC-F | ATGAAACGTCAAAAACGATTTGAAGAA | This study |
|  | prLCH-PduC-R | CAGTTCCAAATGTTCTGGATCAAGGGC | This study |
|  | prLCH-PduC-F313 | ATTACATCAGCTTTGACTCCTGC | This study |
|  | prLCH-PduC-R1611 | GTCTTCATATAGACGGTATCCAGT | This study |
|  | prLCH-PduC-MAMA-R302 | ATACA**GGC**TACGGCACCATTTTGA | This study |
|  | prLCH-PduC-MAMA-R337 | ATGC**TGC**GTCACAACCAGAAGCACAT | This study |
|  | oLCH1 | GGAACAGCACCAGGAATTTCAATACA**GGC**TACGGCACCATTTTGAAGTCCT | This study |
|  | oLCH2 | CGCATATCGGAGTGTGAGTATGC**TGC**GTCACAACCAGAAGCACATTCGATG | This study |
