# Supplementary material for: Exploring Lactobacillus reuteri DSM20016 as a biocatalyst for transformation of longer chain 1,2-diols: Limits with microcompartment
Source: PLoS One. 2017 Sep 28;12(9):e0185734. doi: 10.1371/journal.pone.0185734 (PMC5619818; doi:10.1371/journal.pone.0185734)
Supplement: S2 Table — (DOCX) [file pone.0185734.s005.docx]

| **Organisms** | **Location** | **Gene length (bp)** | | | **Protein molecular mass (kDa)** | | |
| --- | --- | --- | --- | --- | --- | --- | --- |
|  |  | α | β | γ | α | β | γ |
| ***Clostridium carboxidivorans P7*** | *MCP* | 1665 | 669 | 513 | 60,774 | 24,038 | 19,084 |
| ***Clostridium carboxidivorans P7*** | *MCP* | 1665 | 666 | 513 | 60,745 | 23,836 | 19,273 |
| ***Citrobacter freundii CFNIH1*** | *MCP* | 1665 | 675 | 519 | 60,253 | 24,320 | 19,234 |
| ***Citrobacter freundii CFNIH1*** | *-* | 1668 | **585** | **429** | 60,604 | **21,389** | **16,103** |
| ***Citrobacter freundii CFNIH1*** | *-* | 2433 | - | - | 90,113 | - | - |
| ***Klebsiella pneumoniae subsp. pneumoniae HS11286*** | *MCP* | 1665 | 690 | 525 | 60,324 | 24,484 | 19,556 |
| ***Klebsiella pneumoniae subsp. pneumoniae HS11286*** | *-* | 1668 | **585** | **426** | 60,660 | **21,341** | **16,104** |
| ***Klebsiella pneumoniae subsp. pneumoniae HS11286*** | *-* | 2433 | - | - | 90,324 | - | - |
| ***Clostridium pasteurianum DSM 525*** | *-* | 1665 | **540** | **441** | 60,813 | **19,549** | **16,722** |
| ***Clostridium perfringens ATCC 13124*** | *-* | 1665 | **567** | **426** | 60,996 | **20,624** | **16,283** |
| ***Clostridium butyricum strain KNU-L09*** | *-* | 2364 | - | - | 88,047 | - | - |
| ***Terrisporobacter glycolicus ATCC14880*** | *MCP* | 2544 | - | - | 94,332 | - | - |
| ***Enterococcus malodoratus ATCC 43197*** | *MCP* | 1662 | 666 | 513 | 60,301 | 23,882 | 18,668 |
| ***Klebsiella oxytoca strain CAV1374*** | *MCP* | 1665 | 675 | 522 | 60,348 | 24,079 | 19,173 |
| ***Listeria monocytogenes EGD-e*** | *MCP* | 1665 | 660 | 513 | 60,439 | 23,973 | 18,992 |
| ***Streptococcus sanguinis SK36*** | *MCP* | 1662 | 669 | 516 | 60,703 | 24,110 | 19,072 |
| ***Salmonella enterica subsp.*** | *MCP* | 1665 | 675 | 522 | 60,362 | 24,173 | 19,144 |
| ***Yersinia enterocolitica subsp.*** | *MCP* | 1665 | 672 | 525 | 60,192 | 24,175 | 19,040 |
| ***Lactobacillus brevis ATCC 367*** | *MCP* | **1677** | **720** | 528 | **61,429** | **25,690** | 19,362 |
| ***Lactobacillus reuteri DSM 20016*** | *MCP* | **1677** | **711** | 516 | **62,092** | **25,808** | 19,188 |
| ***Lactobacillus collinoides DSM 20515*** | *MCP* | **1677** | **708** | 528 | **61,710** | **25,498** | 19,649 |
